# Serum Alpha-Fetoprotein Predicts Treatment Outcome in Chronic Hepatitis C Patients Regardless of HCV Genotype

**DOI:** 10.1371/journal.pone.0002391

**Published:** 2008-06-11

**Authors:** Hendy Abdoul, Vincent Mallet, Stanislas Pol, Arnaud Fontanet

**Affiliations:** 1 Unité d'Épidémiologie des Maladies Émergentes, Institut Pasteur, Paris, France; 2 Université Paris Descartes, APHP, Hôpital Cochin, Unité d'hépatologie, INSERM U.567, Paris, France; University of California San Francisco, United States of America

## Abstract

We examined the association between serum alpha-fetoprotein (AFP) level and sustained virological response (SVR) in 93 chronic hepatitis C patients. The SVR rate was much higher among patients with serum AFP levels below rather than above the median value (5.7 ng/ml) (58.7% and 19.2%, respectively; P<0.0001). Serum AFP should be added to the list of factors predictive of treatment response in chronic hepatitis C.

## Introduction

Serum alpha-fetoprotein (AFP) is a fetal glycoprotein produced by the yolk sac and fetal liver [1]. Following birth, AFP levels decrease rapidly to less than 20 ng/ml and increase significantly in certain pathologic conditions. Serum AFP is a debated, but routinely used marker for hepatocellular carcinoma (HCC) in patients with chronic liver disease [2]. Yet, significant elevations of AFP are commonly seen in non-hepatic malignancies and benign conditions, such as acute and chronic viral hepatitis [3].

In a previous study [4], we have demonstrated that AFP was negatively associated with treatment response in 100 patients with chronic hepatitis C in Egypt. Indeed, the sustained virological response (SVR) rate was 40.8% among patients with AFP above the median value (4.5 ng/mL), and 80.4% for patients with AFP below the median value (P<0.001). Adjustment for other factors associated with SVR (age, gender, body mass index, steatosis score, fibrosis stage, ALT level, hemoglobin level, clotting time, HCV RNA viral load, and treatment dose received) did not affect the relationship. One limitation of the study was that patients were almost all (99%) infected with HCV genotype 4, and hence results may not be applicable to other genotypes. In this paper, we present the results of a similar study performed in France among patients infected with other HCV genotypes.

## Methods

### Participants

This retrospective study included patients with chronic hepatitis C who attended the Liver Unit at Necker Hospital, Paris, during 2002, and in whom antiviral therapy was initiated during this period. The inclusion criteria for antiviral therapy were: age over 18 years, positive serology for HCV antibodies, positive HCV RNA, and a pathological METAVIR score strictly higher than A1F1. Exclusion criteria were classic contraindications to interferon or ribavirin therapy, including autoimmune disorders, pregnancy, limiting psychiatric, ophthalmological or cardiological disorders, platelet count less than 75,000/mm^3^, neutrophil counts less than 1500 cells/mm^3^, and haemoglobin levels less than 10 g/dl. Child-Pugh C cirrhosis, and positive hepatitis B antigen were also excluded. Antiviral therapy included pegylated interferon alpha-2b (PEG intron, Schering Plough) and ribavirin (Rebetol, Schering Plough), initially given as a function of the patient's weight. The initial dosage was always higher than 1 µg/kg/week subcutaneously for pegylated interferon alpha-2b and 10.6 mg/kg/day orally for ribavirin. The duration of treatment was defined according to consensus recommendations according to genotype, HCV viral load, presence of cirrhosis and failure of previous treatment. All patients were followed for at least 6 months after treatment discontinuation in order to assess their SVR. HCV RNA was measured in the serum using the reverse PCR (COBAS™ Amplicator™, Roche). Genotyping was performed using the Inno-Lipa assay (Innogenetics, Ghent, Belgium). Hepatic necroinflammation and fibrosis were assessed by the METAVIR scoring system [5]. Fibrosis was staged on a scale of F0–F4: F0 = no fibrosis, F1 = portal tract expansion by fibrosis, F2 = less than 50% bridging fibrosis, F3 = more than 50% bridging fibrosis without cirrhosis, and F4 = established cirrhosis. Steatosis was staged as follows: 0: no steatosis, 1: 1–29% of hepatocytes, 2: 30–49% of hepatocytes and 3: ≥50% of hepatocytes.

### Ethics

This study is based on the retrospective analysis (performed in 2007) of clinical and laboratory data collected in 2002 as part of the routine management of patients with chronic hepatitis C in the Liver Unit at Necker Hospital, Paris, France. A specific study code number was created for each patient so that no name or identifier other than age and sex appears in the database. Neither informed consent nor ethics approval was asked for this analysis, since it is exclusively retrospective and did not require any additional testing other than that performed as part of routine management of patients in 2002.

### Statistical methods

The initial database contained 104 individuals. Those who were infected with genotype 4 (n = 4), and those with HIV co-infection (n = 7), were excluded from the analysis so that the assessment of AFP predictive value would be carried out only in HIV-negative non genotype 4-infected individuals. Most continuous variables were categorised according to medians or quartiles, except for body mass index (≤ or >25 kg/m2), platelets (< or ≥150,000/mm3), AST (< or ≥25 and 30 UI/L for females and males, respectively) and ALT (< or ≥25 and 30 UI/L for females and males, respectively). Factors associated with SVR were examined in univariate and multivariate logistic regression. Factors retained in the final multivariate regression model all had P values less than 0.05, except for AFP which was forced into the model. Likelihood ratio tests were used to determine P values.

All analyses were performed using Stata 9.0 statistical software (Stata Corporation, College Station, TX, USA).

## Results

Baseline clinical, biological, virological and histological characteristics of the participants are given in Table 1. The majority (58.1%) of patients were males, with median age of 48 years. Seventy-three (78.5%) had significant (≥F2) fibrosis, and 52 (55.9%) had severe (≥F3) fibrosis. Infecting HCV genotypes were 1 (62.4%), 2 (7.5%), 3 (27.9%), 5 (1.1%) and 6 (1.1%). Median (IQR) serum AFP level was 5.7 (2.8–9.9) ng/ml, with values ranging from 1.2 to 103 ng/ml.

**Table 1:** Relationship between socio-demographical, biological and histological variables and SVR among 93 chronic hepatitis C patients treated with combined therapy.

Variable	n (%)	Number of SVR (%)	Univariate OR (95% CI)	p	Multivariate^1^ OR (95% CI)	p
Gender						
Female	39 (41,9)	20 (51,3)	1,00			
Male	54 (58,1)	16 (29,6)	0,40 (0,17–0,94)	0,036		
Age (year)						
≤42	25 (26,9)	14 (56,0)	1,00			
42–48	22 (23,7)	6 (27,3)	0,29 ( 0,09–1,00)			
48–56	22 (23,7)	9 (40,9)	0,54 (0,17–1,73)	0,161		
>56	24 (25,8)	7 (29,2)	0,32 (0,10–1,05)			
BMI (kg/m2)						
≤25	43 (51,2)	18 (41,9)	1,00			
>25	41 (48,8)	15 (36,6)	0,80 (0,33–1,93)	0,621		
Creatinin (micromol/L)						
≤83	47 (52,8)	17 (36,2)	1,00			
>83	42 (47,2)	16 (38,1)	1,08 (0,46–2,57)	0,851		
Hemoglobin (g/dL)						
≤14,4	48 (52,2)	27 (56,3)	1,00			
>14,4	44 (47,8)	9 (20,5)	0,20 (0,08–0,51)	0,001		
AST (IU/L)						
<25 (female) or 30 (male)	12 (12,9)	6 (50,0)	1,00			
≥25 or 30	81 (87,1)	30 (37,0)	0,59 (0,17–1,99)	0,390		
ALT (IU/L)						
<25 (female) or 30 (male)	7 (7,5)	4 (57,1)	1,00			
≥25 or 30	86 (92,5)	32 (37,2)	0,44 (0,09–2,11)	0,298		
GGT (IU/L)						
< = 72	46 (49,5)	29 (63,0)	1,00		1,00	
>72	47 (50,5)	7 (14,9)	0,10 (0,04–0,28)	0,0001	0,14 (0,04–0,56)	0,005
Alkalin Phosphatase (IU/L)						
< = 84	48 (51,6)	21 (43,8)	1,00			
>84	45 (48,4)	15 (33,3)	0,64 (0,28–1,49)	0,304		
Platelets (x103/mm3)						
≥150	54 (60,0)	27 (50,0)	1,00			
<150	36 (40,0)	8 (22,2)	0,29 (0,11–0,74)	0,008		
Total Bilirubin (mg/dL)						
≤10	40 (51,3)	16 (40,0)	1,00			
>10	38 (48,7)	14 (36,8)	0,88 (0,35–2,18)	0,775		
Viral load (×103 IU/ml)						
≤475	43 (50,6)	17 (39,5)	1,00			
>475	42 (49,4)	15 (35,7)	0,85 (0,35–2,04)	0,716		
AFP (ng/mL)						
≤2,8	23 (24,7)	16 (69,6)	1,00		1,00	
2,8–5,7	23 (24,7)	11 (47,8)	0,40 (0,12–1,34)		0,66 (0,16–2,77)	
5,7–9,9	23 (24,7)	4 (17,4)	0,09 (0,02–0,37)	0,001	0,51 (0,09–2,80)	0,854
>9,9	24 (25,8)	5 (20,8)	0,11 (0,03–0,43)		0,84 (0,13–5,44)	
Steatosis score						
0	11 (14,5)	7 (63,6)	1,00			
1	39 (51,3)	11 (28,2)	0,22 (0,05–0,92)			
2	17 (22,4)	5 (29,4)	0,24 (0,05–1,19)			
3	9 (11,8)	1 (11,1)	0,07 (0,01–0,80)	0,065		
missing	16					
Adequate^2^ dose of treatment						
No	42 (46,2)	16 (38,1)	1,00			
Yes	49 (53,8)	18 (36,7)	0,94 (0,40–2,21)	0,894		
Fibrosis score (METAVIR)						
≤2	41 (44,1)	28 (68,3)	1,00		1,00	
>2	52 (55,9)	8 (15,4)	0,08 (0,03–0,23)	0,0001	0,18 (0,05–0,60)	0,005
Genotype						
Genotype 2,3,5,6	35 (37,6)	19 (54,3)	1,00		1,00	
Genotype 1	58 (62,4)	17 (29,3)	0,35 (0,14–0,84)	0,018	0,26 (0,08–0,87)	0,029

1 Multivariate analysis includes GGT, AFP, Fibrosis score and genotype

OR, odds-ratio; CI, confidence interval, AFP, alpha-foetoprotein; GGT, serum gamma glutamyl transpeptidase

2 Dose of treatment was considered adequate if patients had received at least 80% of the intended dose for at least 80% of the duration of treatment

The SVR rate was 36/93 (38.7%), and decreased with increasing levels of serum AFP: 69.6%, 47.8%, 17.4%, and 20.8%, for the 1st, 2nd, 3rd, and 4th quartiles, respectively (P = 0.001). Also, the SVR rate was 58.7% and 19.2% for those with serum AFP under and above the median value, respectively (OR = 0.17, 95% CI = 0.06–0.42; P<0.0001). For patients infected with genotype 1, SVR rates for patients with fibrosis scores of 0–2 (low to moderate) and 3–4 (severe) were 54.5% and 13.9%, respectively. For patients infected with other genotypes (2,3,5, and 6), the SVR rates for the same fibrosis categories were 84.2% and 18.8%, respectively.

Other factors associated with SVR in univariate analysis were gender, haemoglobin level, serum AST, serum gamma-glutamyl transpeptidase (GGT), and platelet counts (Table 1). In multivariate analysis, only serum GGT above the median level, severe fibrosis, and genotype 1 infection were negatively and independently associated with SVR. AFP was no longer significant in multivariate analysis, after introduction of the variables “severe fibrosis” and “serum GGT” in the model. Of note, serum AFP and GGT were strongly correlated (spearman rank coefficient = 0.61, P<0.0001), and median AFP levels were significantly higher among patients with severe fibrosis compared to others (12.6 versus 4.8 ng/ml, respectively, P = 0.002). There was no interaction between genotypes and serum AFP, i.e., the OR of the association between serum AFP and SVR was no different for patients infected with HCV genotype 1 and others.

## Discussion

This study confirms our previous findings regarding the predictive role of AFP with regard to treatment response in chronic hepatitis C. In this study, the odds of treatment failure was six times higher for those with serum AFP above the median value (5.7 ng/ml), a figure identical to that observed in our previous study among Egyptian patients infected with HCV genotype 4 (OR = 5.9; median value 4.5 ng/ml) [4]. This effect is far from negligible, and of the same magnitude than that of other well-accepted predictive factors of treatment response such as HCV genotype. Of interest, patients in this study were infected with various genotypes, the majority being genotype 1, thus confirming that our initial observation was not genotype 4-specific. Based on these results, clinicians should take serum AFP results into account in their evaluation of patient's likelihood of responding to treatment.

The multivariate analysis provides us with additional information on the mechanisms by which serum AFP might be associated with treatment response. One difference with our previous study is the disappearance of the association between AFP and SVR in multivariate analysis, after controlling for both serum GGT and liver fibrosis. These two factors have been shown associated with lower treatment response rates elsewhere [6], [7]. In our previous study among Egyptian patients, serum AFP remained independently associated with SVR after controlling for known factors associated with SVR, including liver fibrosis. In that study, it was the association between liver fibrosis and SVR which was completely removed after introducing serum AFP level into the model. Thus, there seems to be important correlations among liver fibrosis, serum AFP, and serum GGT, one acting as a confounder of the other in the association with SVR depending on the study population. Hepatic progenitor cells (HPC) may be the unifying factor explaining these observations. HPC arise in the periportal region of the liver and may be responsible for liver regeneration. They express high levels of AFP, certain keratin markers, and GGT [8]–[10]. Their presence is related to the severity of fibrosis [11], and their activation has been documented in parallel with cells associated with the development of fibrosis (stellate cells) [12]. Hence, the joint association observed in this study among AFP, GGT, liver fibrosis and SVR may be the reflection of more intense HPC expression in non responders compared to responders. Quite interestingly, HPC expression has recently been associated with response to treatment, being higher in non-responders and relapsers compared with responders [11].

In conclusion, this study confirms the value of serum AFP levels in predicting treatment outcome in patients with chronic hepatitis C, regardless of the infecting genotype. Higher levels of serum AFP may correspond to higher expression of HPC in individuals developing liver fibrosis.
